# EAGLE-DET: Edge-Aware Global–Local Enhancement for Small Object Detection in UAV Aerial Imagery

**DOI:** 10.3390/s26113554

**Published:** 2026-06-03

**Authors:** Yimeng Tao, Yan Ding, Bo Mo, Bozhi Zhang, Chunbo Zhao, Dawei Li

**Affiliations:** 1 School of Aerospace Engineering, Beijing Institute of Technology, Beijing 100081, China; 3120235052@bit.edu.cn (Y.T.); mobobit@163.com (B.M.); 18865515859@163.com (C.Z.); 2 Beijing Special Machinery Research Institute, Beijing 100089, China; zhangbozhi93@126.com; 3 Southwest Institute of Technical Physics, Chengdu 610041, China; 15359312805@163.com

**Keywords:** UAV object detection, small object detection, edge enhancement, multi-scale feature fusion, sparse attention mechanism

## Abstract

Small object detection in UAV aerial imagery poses significant challenges due to sparse pixel representation and ambiguous object boundaries. Through systematic analysis, we identify three critical degradation stages during forward propagation in deep detection networks: edge attenuation during feature extraction, semantic conflict during feature fusion, and detail loss during feature reconstruction. Existing methods address these stages in isolation or implicitly, lacking collaborative and stage-aware repair strategies. To address this issue, we propose EAGLE-DET, a novel detection framework based on sparse multi-scale attention and refined transformation. Specifically, the framework comprises three core modules: (1) the Cross-stage Multi-resolution Edge Enhancement Network (CMENet), which preserves small object edge representations via adaptive high-low frequency decomposition; (2) the Attention-guided Multi-scale Feature Fusion Network (AMFFN), which resolves cross-scale semantic conflicts through pyramidal sparse attention and multi-scale spatial decoupling; (3) the Enhanced Upsampling with Channel Bridging and Spatial Coordination module (EUCBSC), which recovers spatial detail fidelity via bidirectional channel shift mixing. Extensive experiments on three benchmark datasets—VisDrone-2019, UAVDT, and DOTA1.0—demonstrate the effectiveness of EAGLE-DET, which achieves improvements of 4.5% AP50 and 2.9% AP50:95 on VisDrone-2019 over the baseline, while maintaining inference at 71.7 FPS, achieving an optimal accuracy–efficiency trade-off.

## 1. Introduction

**Figure 1:**
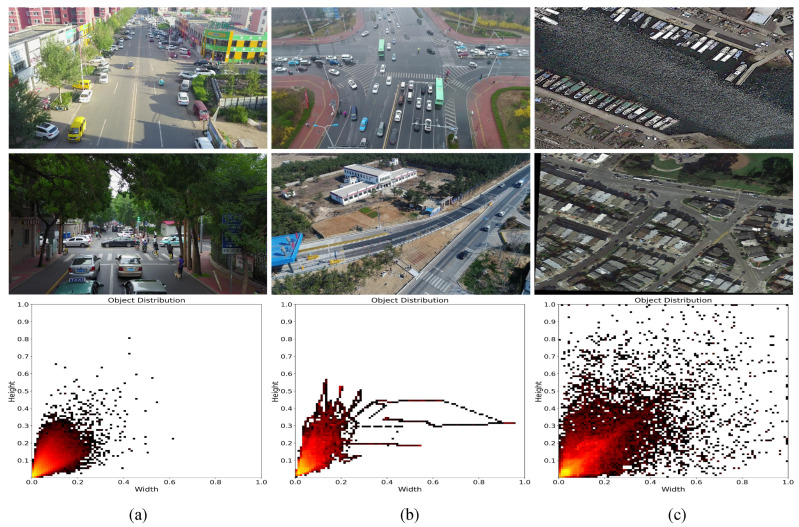
Example images and object size distribution of three representative UAV aerial datasets. (**a**) The VisDrone-2019 dataset. (**b**) The UAVDT dataset. (**c**) The DOTA1.0 dataset.

Unmanned aerial vehicle (UAV)-based object detection has emerged as crucial technology with diverse applications, including traffic monitoring, disaster rescue, and urban surveillance [1]. Unlike ground-view imagery, UAV aerial images exhibit wide fields of view and flexible acquisition, but impose severe detection challenges: objects captured from high altitudes typically occupy only tens of pixels, possess blurred boundaries, and appear densely distributed against complex backgrounds. According to the MS COCO benchmark [2], small objects are formally defined as objects with pixel areas smaller than 32^2^ pixels. As shown in Figure 1, small objects dominate in three representative UAV aerial datasets: VisDrone-2019 [3], UAVDT [4], and DOTA1.0 [5], making small object detection a central bottleneck for UAV vision systems.

Deep convolutional neural networks have achieved remarkable success in general object detection tasks, but face fundamental challenges of insufficient feature representation in aerial small object detection scenarios. Researchers have proposed improvement schemes from different perspectives. Swin Transformer [6] enhances global feature modeling capability through hierarchical window attention mechanisms, but has high computational complexity and lacks explicit modeling of edge information. ConvNeXt [7] and RepViT [8] achieve good balance between efficiency and accuracy, but still show inadequacy in small object edge feature extraction. BiFPN [9] achieves more flexible multi-scale fusion through bidirectional feature pyramids and learnable weights, while AFPN [10] introduces progressive feature fusion strategies, but these methods essentially still rely on linear fusion paradigms, lacking deep modeling of cross-scale feature semantic alignment. CARAFE [11] proposes content-aware feature reorganization strategies, and DySample [12] designs dynamic sampling mechanisms, but these methods mainly focus on feature recovery in the spatial dimension, neglecting the important role of inter-channel information interaction in small object feature enhancement. More critically, existing research lacks systematic understanding and collaborative solutions for the degradation problem of small objects in network forward propagation.

This paper identifies three critical degradation stages: First, edge attenuation during the feature extraction stage [13]. Successive downsampling in backbone networks progressively attenuates high-frequency edge information that small objects critically depend on. Traditional residual architectures lack stage-aware protection mechanisms for such edge features, causing them to weaken or vanish in deeper layers. Second, semantic conflict during the feature fusion stage [9,14]. When fusing features across large-scale spans, simple linear concatenation or element-wise addition cannot resolve the substantial gap in semantic abstraction levels between shallow and deep feature maps, generating information redundancy that masks critical small object features. Third, conventional upsampling operators like bilinear and nearest-neighbor blend target edge pixels with background during resolution recovery, introducing aliasing artifacts that dilute the spatial structural integrity of small objects [11,12]. Crucially, these three stages are sequential and mutually compounding: unaddressed degradation at each stage amplifies errors in subsequent stages.

We propose EAGLE-DET, which addresses the three-stage degradation through cascaded feature repair. For edge attenuation, the CMENet backbone employs the Cross-Stage Pyramidal Multi-Resolution Edge Enhancement (CSPMEE) module to construct multi-scale feature pyramids, with the EdgeEnhancer sub-module providing adaptive edge enhancement. For semantic conflict, the Attention-guided Multi-scale Feature Fusion Network (AMFFN) employs the Hierarchical Sparse Attention Transformer (HSAT) to establish long-range cross-scale semantic associations via pyramidal sparse attention, combined with Spatial Decomposition Enhanced Convolution (SDEC) for multi-scale spatial decoupling of shallow features, thereby suppressing background noise. For detail loss, the Enhanced Upsampling with Channel Bridging and Spatial Coordination module (EUCBSC) integrates depth-wise separable convolution, channel shuffle, and SDEC to preserve edge sharpness and structural integrity during resolution recovery. The three modules form a cascaded enhancement chain from edge-aware extraction to semantic-aligned fusion to high-fidelity reconstruction. The main contributions of this paper are summarized as follows:

- First, a theoretical analysis framework for three-stage degradation of small object features is proposed, providing a new theoretical perspective for the design of aerial small object detection algorithms;
- Second, the CMENet, including cross-stage pyramidal structure and multi-resolution edge enhancement modules, is proposed to handle the edge feature attenuation problem;
- Third, the AMFFN is proposed for the adaptive semantic alignment of cross-scale features through a pyramidal sparse attention mechanism and a multi-scale spatial decoupling strategy;
- Fourth, the EUCBSC, which enhances feature reconstruction quality through a bidirectional channel-shift mixing mechanism, is proposed to alleviate the upsampling detail loss problem.

The remainder of this paper is organized as follows: Section 2 reviews related work; Section 3 details the proposed EAGLE-DET algorithm; Section 4 presents experimental results and analysis. Section 5 discusses the model proposed in this paper and Section 6 concludes the paper.

## 2. Related Work

### 2.1. UAV Small Object Detection

Object detection has evolved from two-stage detectors to one-stage detectors and then to end-to-end Transformer detectors. Based on mature object detection frameworks, researchers have proposed numerous specialized algorithms for UAV small object detection. Within the UAV domain, TPH-YOLOv5 [15] incorporates self-attention into YOLOv5 to handle dense scenes; QueryDet [16] employs cascaded sparse queries for computational efficiency; DMF-YOLO [17] designs dynamic multi-scale fusion for small object enhancement. RT-DETR [18], which serves as our baseline, achieves real-time end-to-end detection by eliminating NMS and using IoU-aware query selection. Subsequent works further adapt RT-DETR for UAV scenarios: Sparse-DETR [19] introduces sparse attention mechanisms and progressive query selection strategies. Drone-DETR [20] introduces lightweight backbones and shallow feature enhancement, while UAV-DETR [14] integrates multi-scale fusion with spatial alignment. However, a careful examination reveals that each method addresses only a subset of the degradation stages identified in this work. Specifically, Drone-DETR [20] improves the backbone and shallow feature enhancement, primarily targeting edge attenuation during extraction, but does not address semantic conflict in fusion or detail loss in reconstruction. UAV-DETR [14] combines multi-scale fusion with spatial alignment, partially alleviating semantic conflict, yet treats backbone extraction and upsampling as independent components without considering their compounding effects. DMF-YOLO [17] designs dynamic multi-scale fusion for small object enhancement, focusing exclusively on the fusion stage while lacking edge-aware extraction and high-fidelity reconstruction. In contrast, EAGLE-DET provides a unified three-stage framework where each module is explicitly designed to repair a specific degradation stage, and the modules are cascaded to prevent error propagation across stages.

### 2.2. Feature Extraction Networks

ResNet [21] established the residual connection paradigm for deep feature extraction, but its fixed architecture lacks adaptive mechanisms for small object edge preservation. FasterNet [22] proposes partial convolution (PConv) for computational efficiency. Gold-YOLO [23] achieves multi-scale aggregation through Gather-and-Distribute. In network architecture design, InternImage [24] constructs powerful feature extraction networks using deformable large kernel convolutions, while UniRepLKNet [25] proposes a unified architectural design paradigm for large kernel convolutions, achieving excellent performance in multiple vision tasks. Vision Transformer [26] introduces self-attention mechanisms into vision tasks, demonstrating powerful global modeling capability, but has high computational complexity. ConvNeXt [7] revisits the design space of convolutional networks, bringing pure convolutional architectures to performance comparable to Transformers through modernization. MambaVision [27] combines Mamba with Transformer architectures, enhancing global feature modeling capability while maintaining efficient computation. Edge information is crucial for small object detection, as discriminative features of small objects greatly depend on their contour shapes. PiDiNet [28] efficiently extracts edge features through pixel-difference convolution. DiffusionEdge [29] explores the application potential of diffusion models in edge detection. However, these methods are typically used as independent modules, lacking deep integration with the feature extraction process of backbone networks. Existing backbone networks lack explicit modeling of edge high-frequency information, with continuous downsampling causing layer-by-layer attenuation of small object edge features.

### 2.3. Multi-Scale Feature Fusion and Reconstruction

Multi-scale feature fusion aims to comprehensively utilize semantic information and spatial details from features at different levels. FPN [30] pioneered the top-down feature pyramid for multi-level fusion, subsequently extended by PANet [31] with bottom-up path aggregation, BiFPN [9] with bidirectional pyramids and learnable weights, and AFPN [10] with progressive non-adjacent layer interaction. Attention-based fusion methods include DANet [32] with parallel position-channel attention, BiFormer [33] with bi-level routing attention, and SCSA [34] which explores spatial-channel synergy. In upsampling, CARAFE [11] generates content-aware kernels, SAPA [35] employs similarity-aware point affinity, and DySample [12] learns dynamic sampling patterns. However, these methods mainly focus on single-scale feature enhancement, showing inadequacy in modeling semantic alignment among cross-scale features. Linear fusion paradigms struggle to effectively model semantic correspondence relationships among cross-scale features, easily producing semantic conflicts when fusing features with large-scale spans.

In feature reconstruction, upsampling operations determine the quality of deep semantic features recovering to high resolution. Bilinear interpolation, as the most commonly used upsampling method, achieves resolution improvement through weighted averaging, but introduces edge blur and aliasing artifacts. Deconvolution [36] achieves upsampling through learnable parameters, but easily produces checkerboard effects. CARAFE [11] proposes content-aware feature reorganization strategies, dynamically generating upsampling kernels based on input content. SAPA [35] achieves high-quality feature upsampling through similarity-aware point affinity modeling. These methods improve upsampling quality to some extent, but mainly focus on feature recovery in the spatial dimension, neglecting the potential role of inter-channel information interaction in small object feature enhancement. Our proposed approach differs from these existing methods in two key aspects. First, unlike BiFPN [9] and AFPN [10] that rely on linear fusion paradigms, our AMFFN integrates pyramidal sparse attention with spatial decoupling convolution, enabling adaptive semantic alignment across scales through learnable cross-scale correspondences rather than fixed weighted summation. Second, unlike CARAFE [11] and DySample [12], which operate solely in the spatial dimension, our EUCBSC combines spatial resolution recovery with inter-channel information mixing through bidirectional channel shift, simultaneously enhancing both spatial fidelity and channel-wise feature diversity during reconstruction.

## 3. Method

**Figure 2:**
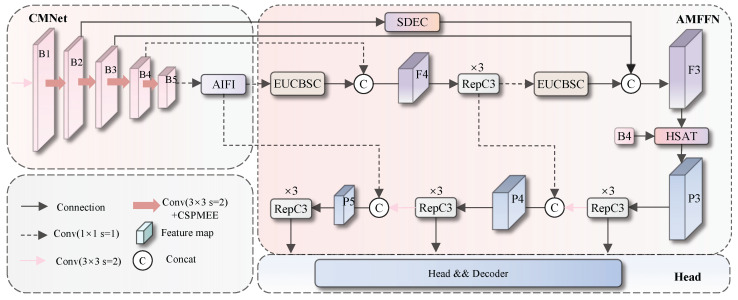
Overall architecture of EAGLE-DET. The framework consists of three core modules: a CMENet backbone network for edge-aware feature extraction, AMFFN for attention-guided multi-scale feature fusion, and EUCBSC for enhanced upsampling with channel-spatial coordination. The modules work collaboratively to address the three-stage degradation problem of small object features.

We propose EAGLE-DET, an end-to-end detection framework that instantiates a dedicated repair strategy at each of the three identified degradation stages. The overall architecture is shown in Figure 2. Specifically, the Cross-stage Multi-resolution Enhancement Network (CMENet) provides multi-scale edge-enhanced feature maps to an Attention-guided Multi-scale Feature-Fusion Network (AMFFN), which performs semantically aligned cross-scale fusion; the fused features are then passed to an Enhanced Upsampling with Channel Bridging and Spatial Coordination module (EUCBSC) for high-fidelity upsampling before entering the detection head. The three modules construct a complete feature enhancement chain from edge-aware extraction to semantic-aligned fusion to high-fidelity reconstruction, systematically solving the degradation problem of small object features during network forward propagation. For clarity, a comprehensive summary of all mathematical symbols and notations used in this section is provided in Appendix A.

### 3.1. Cross-Stage Pyramidal Multi-Resolution Edge Enhancement Backbone Network Design

**Figure 3:**
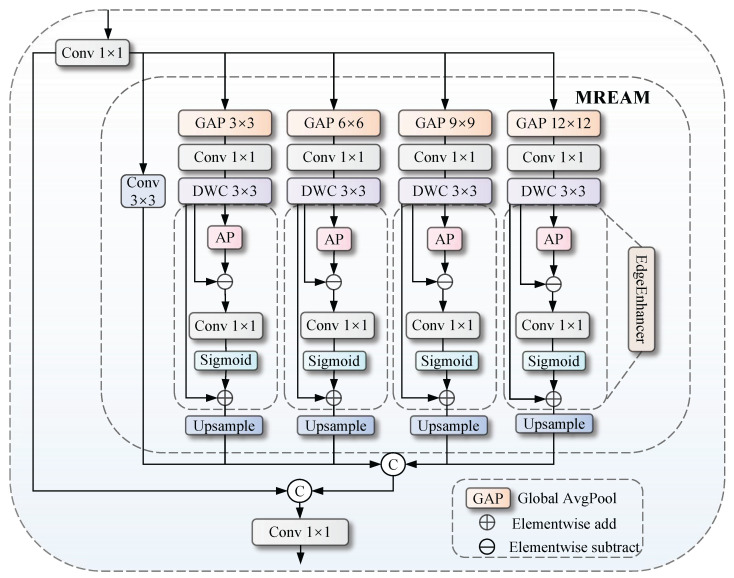
Architecture of the CSPMEE module in the CMENet backbone network. The CSPMEE module adopts a branch-fusion feature-processing paradigm, consisting of bypass and enhancement branches. The enhancement branch contains MREAM units that construct multi-scale feature pyramids through parallel adaptive pooling branches with different scales. Each branch incorporates EdgeEnhance for adaptive edge enhancement based on high-frequency and low-frequency decomposition.

The CMENet backbone, built on Cross-Stage Pyramidal Multi-Resolution Edge Enhancement (CSPMEE) modules, targets edge attenuation during feature extraction. The structure of CSPMEE is shown in Figure 3. It combines the efficiency of cross-stage partial connections with multi-scale edge enhancement, improving small object edge perception while maintaining a lightweight design.

The CSPMEE module adopts a branch-fusion feature processing paradigm, achieving balanced distribution of computational load by decomposing input feature maps along the channel dimension into multiple subspaces. The module first uses 1×1 convolution to map input features to an intermediate representation with twice the number of channels, then uniformly divides along the channel dimension into two branches, where the reserved branch maintains the structural integrity of original features and the enhancement branch performs deep-feature transformation through concatenated Multi-Resolution Edge Amplification Module (MREAM) units. The mathematical expression of the entire module can be formalized as

(1) $$F_{\mathrm{CSPMEE}}\left(X\right)=\psi_{1\times 1}\;\left(C\;\left[X_{\mathrm{bypass}},\;\overset{n}{\underset{i=1}{⋂}}M_{\mathrm{MREAM}}^{\left(i\right)}\left(X_{\mathrm{branch}}\right)\right]\right)$$

where $X_{\mathrm{bypass}}$ and $X_{\mathrm{branch}}$ represent feature tensors of bypass and enhancement branches respectively, $M_{\mathrm{MREAM}}^{\left(i\right)}$ represents the *i*-th MREAM unit, C[·] denotes feature concatenation operation, and $\psi_{1\times 1}$ is the final 1×1 convolution mapping function. In our implementation, the number of cascaded MREAM units *n* is set to 1 for all CSPMEE stages. After the initial 1×1 convolution, the tensor is split equally along the channel axis into $X_{\mathrm{bypass}}$ and $X_{\mathrm{branch}}$, each with half the channels. After the MREAM unit, the two branches are then concatenated and projected back to the original channel count via the final 1×1 convolution.

The MREAM module constructs a hierarchical multi-scale feature pyramid, capturing feature representations under different spatial receptive fields through parallel adaptive pooling branches. The module designs four different scale pooling kernels {3×3,6×6,9×9,12×12}. Each branch independently performs feature transformation and then edge information enhancement through EdgeEnhance, finally fusing with local convolution branches to form comprehensive feature representation. The mathematical description of multi-scale feature extraction is

(2) $$F_{\mathrm{multi}}^{\left(k\right)}\left(X\right)=I\;\left(E\;\left(D_{3\times 3}\;\left(\phi_{1\times 1}\;\left(P_{k\times k}^{\mathrm{adaptive}}\left(X\right)\right)\right)\right),\;H\times W\right)$$

where $P_{k\times k}^{\mathrm{adaptive}}$ represents adaptive average pooling of scale k×k, $\phi_{1\times 1}$ is 1×1 convolution for channel compression, $D_{3\times 3}$ denotes 3×3 depth-wise separable convolution, I(·,H×W) is bilinear interpolation upsampling operation, and *E* represents the EdgeEnhance enhancement function. The output of the entire module is obtained through multi-branch feature fusion:

(3) $$F_{\mathrm{MREAM}}\left(X\right)=\psi_{1\times 1}\;\left(C\;\left[\phi_{3\times 3}\left(X\right),\;\underset{k\in \{3,6,9,12\}}{⋃}F_{\mathrm{multi}}^{\left(k\right)}\left(X\right)\right]\right)$$

The EdgeEnhancer sub-module achieves edge enhancement based on high-frequency and low-frequency decomposition edge enhancement theory, extracting edge features through difference operation between smoothing filtering and original signals, and adopting gating mechanisms for adaptive enhancement. Its core computation process is expressed as

(4) $$E\left(X\right)=X+\sigma \;\left(\varphi \;\left(H_{\mathrm{high}}\left(X\right)\right)\right)$$

(5) $$H_{\mathrm{high}}\left(X\right)=X-P_{3\times 3}^{\mathrm{avg}}\left(X\right)$$

where $P_{3\times 3}^{\mathrm{avg}}$ represents a 3×3 average pooling operation for extracting low-frequency smooth components, $H_{\mathrm{high}}$ is the high-pass filtering result, φ represents edge feature transformation convolution, and σ is the Sigmoid gating function. From a frequency-domain perspective, the EdgeEnhancer implements an explicit high-pass filtering operation: the average pooling $P_{3\times 3}^{\mathrm{avg}}$ extracts the low-frequency smooth component of the input signal, and the subtraction operation in Equation (5) isolates the high-frequency residual containing edge and texture information. The subsequent Sigmoid gating mechanism in Equation (4) allows the network to adaptively control the enhancement intensity at each spatial location, selectively amplifying high-frequency edge responses in regions where small objects are present while suppressing noise-dominant high-frequency components in background regions.

This design enables fine-grained edge contours of small objects to be consistently preserved across successive downsampling stages, which directly contributes to improved localization accuracy and category discrimination for small objects in complex aerial scenes.

### 3.2. Attention-Guided Multi-Scale Feature Fusion Network

**Figure 4:**
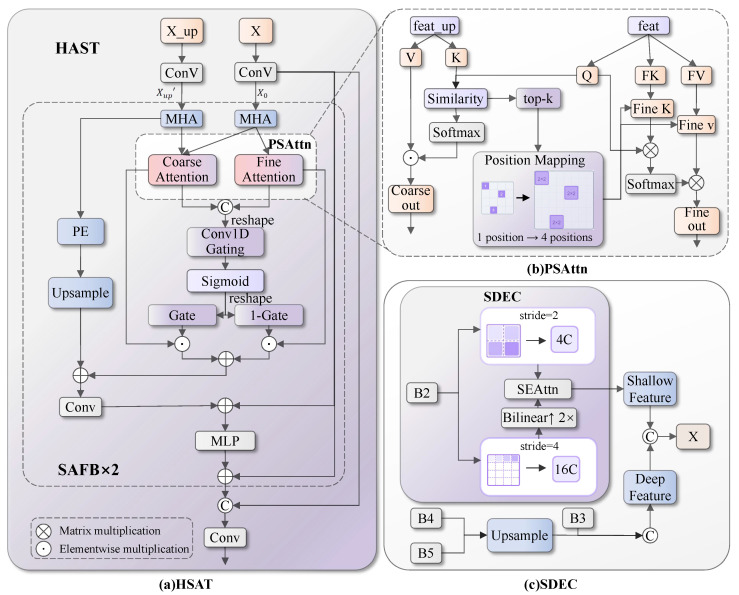
Architecture of AMFFN feature fusion network. The network adopts a hierarchical multi-scale feature interaction architecture through a cascaded combination of SDEC and HSAT. (**a**) The HSAT module. (**b**) Detailed structure of the PSAttn mechanism. (**c**) The SDEC module.

The Attention-guided Multi-scale Feature Fusion Network (AMFFN), whose structure is shown in Figure 4, targets semantic conflict during feature fusion. Through pyramidal sparse attention and multi-scale spatial decoupling, AMFFN adaptively learns cross-scale semantic correspondences while reducing computational complexity and maintaining sensitivity to small object features.

The AMFFN feature fusion network adopts a hierarchical multi-scale feature interaction architecture, achieving efficient cross-scale feature fusion through cascaded combination of Spatial Decomposition Enhanced Convolution (SDEC) and a Hierarchical Sparse Attention Transformer (HSAT). The network first uses the SDEC module to perform multi-scale spatial decomposition on shallow features, extracting local feature representations at different granularities, then establishes long-range semantic associations between deep features and shallow features through the HSAT module, and finally achieves effective fusion of multi-level features through adaptive feature aggregation. The mathematical expression of the entire AMFFN network can be formalized as

(6) $$F_{\mathrm{AMFFN}}\left(X\right)=A_{\mathrm{adaptive}}\;\left(T_{\mathrm{HSAT}}\;\left(S_{\mathrm{SDEC}}\left(X_{\mathrm{shallow}}\right),\;X_{\mathrm{deep}}\right)\right)$$

where $X_{\mathrm{shallow}}$ and $X_{\mathrm{deep}}$ represent shallow and deep feature maps respectively, $S_{\mathrm{SDEC}}$ represents spatial decoupling convolution transformation, $T_{\mathrm{HSAT}}$ represents an HSAT processing function, and $A_{\mathrm{adaptive}}$ is an adaptive feature aggregation operation.

HSAT achieves deep interactive fusion of cross-scale features through a Sparse Attention Fusion Block (SAFB). The HSAT module first performs adaptive adjustment of channel dimensions for input features and upper layer features, then progressively enhances feature representation capability through concatenated attention blocks, and finally generates fused features through feature concatenation and convolution transformation. The overall computation process of HSAT can be expressed as

(7) $$F_{\mathrm{HSAT}}\left(X,X_{\mathrm{up}}\right)=\phi_{1\times 1}\;\left(C\;\left[X_{0},\;\overset{n}{\underset{i=1}{⋂}}B_{\mathrm{SAFB}}^{\left(i\right)}\left(X_{i-1},\;X_{\mathrm{up}}^{\prime }\right)\right]\right)$$

where $X_{0}=\psi_{1}\left(X\right)$ is initial feature transformation, $X_{\mathrm{up}}^{\prime }=\psi_{\mathrm{up}}\left(X_{\mathrm{up}}\right)$ is upper layer feature transformation, $B_{\mathrm{SAFB}}^{\left(i\right)}$ represents the *i*-th SAFB, and $\phi_{1\times 1}$ is the final channel fusion convolution.

SAFB achieves deep transformation of features through residual connected attention mechanisms and multi-layer perceptrons, with a computation process including attention enhancement and nonlinear transformation stages. The mathematical description of this module is

(8) $$B_{\mathrm{SAFB}}\left(X_{0},X_{\mathrm{up}}^{\prime }\right)=X_{0}+M_{\mathrm{MLP}}\;\left(X_{0}+A_{\mathrm{PSAttn}}\left(X_{0},X_{\mathrm{up}}^{\prime }\right)\right)$$

where $A_{\mathrm{PSAttn}}$ represents a pyramidal sparse attention function, and $M_{\mathrm{MLP}}$ is multi-layer perceptron transformation. The pyramidal sparse attention mechanism achieves efficient cross-scale feature alignment through coarse-grained and fine-grained two-level attention computation, with core computation including query-key-value attention and sparse fine-grained attention in two branches:

(9) $$A_{\mathrm{coarse}}=\mathrm{Softmax}\;\left(\frac{Q\cdot K^{T}}{\sqrt{d_{k}}}\right)\cdot V_{\mathrm{up}}$$

(10) $$A_{\mathrm{fine}}=\mathrm{Softmax}\;\left(\frac{Q\cdot K_{\mathrm{topk}}^{T}}{\sqrt{d_{k}}}\right)\cdot V_{\mathrm{topk}}\;\mathrm{if}\;\mathrm{topk}>0$$

where $Q=\phi_{q}\left(X\right)$ is a query matrix, $K=\phi_{k}\left(X_{\mathrm{up}}\right)$ and $V_{\mathrm{up}}=\phi_{v}\left(X_{\mathrm{up}}\right)$ are key and value matrices of upper layer features respectively, and $K_{\mathrm{topk}}$ and $V_{\mathrm{topk}}$ are fine-grained key-value matrices based on sparse selection, fusing coarse and fine two-level attention outputs through gating mechanisms. In our implementation, the multi-head attention operation uses four heads. For training, the top-k selection is disabled so that only coarse attention is computed, reducing training cost; during inference, *k* is set to four to enable fine-grained attention refinement. When fine attention is active, the position mapping expands each selected coarse position to its 2×2 neighborhood, yielding 4×k=16 fine-grained key-value pairs per query. This decomposition thus achieves effective cross-scale semantic alignment at substantially lower cost than full attention, with the coarse stage providing global correspondences and the fine stage refining only the most informative local regions.

The SDEC module achieves enhanced processing of shallow features through multi-scale spatial decoupling and channel attention mechanisms. The module designs two space-depth transformation branches with different strides, respectively capturing local feature patterns at 2× and 4× downsampling, adaptively adjusting feature channel weights through squeeze-excitation mechanisms, and finally generating enhanced feature representations through feature fusion. The complete computation process of SDEC is

(11) $$F_{\mathrm{SDEC}}\left(X\right)=\phi_{3\times 3}\;\left(SE\;\left(C\;\left[S_{\mathrm{SPD}}^{\left(2\right)}\left(X\right),\;I\;\left(S_{\mathrm{SPD}}^{\left(4\right)}\left(X\right)\right)\right]\right)\right)$$

where $S_{\mathrm{SPD}}^{\left(s\right)}$ represents space-depth convolution transformation with stride *s*, I is bilinear interpolation upsampling, SE is squeeze-excitation attention, and $\phi_{3\times 3}$ is the final 3×3 convolution. Space-depth convolution transformation achieves feature space dimensionality reduction and channel expansion through a slice recombination operation:

(12) $$S_{\mathrm{SPD}}^{\left(s\right)}\left(X\right)=\phi_{\mathrm{conv}}\;\left(C\;\left[X[\ldots ,\;i::s,\;j::s]\;|\;i,j\in \{0,1,\ldots ,s-1\}\right]\right)$$

The squeeze-excitation layer learns interdependencies among channels through global average pooling and two-layer fully connected networks, with mathematical representation:

(13) $$SE\left(X\right)=X⊙\sigma \;\left(W_{2}\cdot \delta \left(W_{1}\cdot \mathrm{GAP}\left(X\right)\right)\right)$$

where GAP represents global average pooling, $W_{1}$ and $W_{2}$ are weight matrices for dimensionality reduction and expansion respectively, δ is ReLU activation function, σ is Sigmoid function, and ⊙ denotes element-wise multiplication.

AMFFN achieves adaptive semantic alignment of cross-scale features through the collaborative action of HSAT’s pyramidal sparse attention and SDEC’s spatial decoupling, improving the discriminability and robustness of small object features.

### 3.3. Enhanced Upsampling with Channel-Spatial Coordination Mechanism

**Figure 5:**
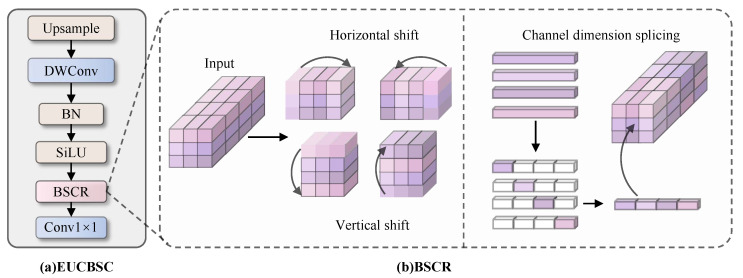
Architecture of EUCBSC. (**a**) EUCBSC module; (**b**) BSCR sub-module.

The Enhanced Upsampling with Channel Bridging and Spatial Coordination module (EUCBSC) targets detail loss during feature reconstruction. The structure of EUCBSC is shown in Figure 5. By integrating depth-wise separable convolution, channel shuffle, and bidirectional channel shift mixing, EUCBSC preserves spatial detail during resolution expansion and enriches feature representation through inter-channel spatial recombination.

The EUCBSC module adopts a cascaded feature enhancement architecture, decomposing the upsampling process into three stages: size expansion, channel recombination, and feature optimization. The module first enlarges the input feature map to target size through bilinear interpolation, then uses depth-wise separable convolution for spatial detail recovery, followed by a channel shuffle mechanism to rearrange feature channels to promote information exchange, and finally performs nonlinear transformation of channel dimensions through point-wise convolution. The mathematical expression of the entire EUCBSC module can be formalized as

(14) $$F_{\mathrm{EUCBSC}}\left(X\right)=\phi_{1\times 1}\;\left(S_{\mathrm{BSCR}}\;\left(S_{\mathrm{shuffle}}\;\left(D_{\mathrm{DW}}\;\left(U_{\mathrm{bilinear}}\left(X,\;s=2\right)\right)\right)\right)\right)$$

where $U_{\mathrm{bilinear}}\left(\cdot ,s=2\right)$ represents a bilinear upsampling operation with scale factor 2, $D_{\mathrm{DW}}$ represents a depth-wise separable convolution transformation, $S_{\mathrm{shuffle}}$ is a channel shuffle function, $S_{\mathrm{BSCR}}$ represents a Bidirectional Spatial Channel Reorganization (BSCR) operation, and $\phi_{1\times 1}$ is a point-wise convolution mapping function.

The BSCR sub-module designs a novel bidirectional channel-shift mixing strategy, enhancing feature spatial diversity and inter-channel information interaction by applying opposite-direction spatial shift operations on different channel groups. The module first uniformly splits the input feature tensor along the channel dimension into four sub-tensors, then applies cyclic shift transformations in horizontal and vertical directions to each sub-tensor respectively. Specifically, the channel splitting operation can be expressed as

(15) $$\left\{X_{1},X_{2},X_{3},X_{4}\right\}=\mathrm{Split}\left(X,\;\dim=1,\;\mathrm{chunks}=4\right)$$

Subsequently, bidirectional shift transformations of sub-tensors are defined as

(16) $$\begin{array}{cc} X_{1}^{\prime } & =\mathrm{Roll}(X_{1},\;\mathrm{shift}=s,\;\dim=H) \end{array}$$

(17) $$\begin{array}{cc} X_{2}^{\prime } & =\mathrm{Roll}(X_{2},\;\mathrm{shift}=-s,\;\dim=H) \end{array}$$

(18) $$\begin{array}{cc} X_{3}^{\prime } & =\mathrm{Roll}(X_{3},\;\mathrm{shift}=s,\;\dim=W) \end{array}$$

(19) $$\begin{array}{cc} X_{4}^{\prime } & =\mathrm{Roll}(X_{4},\;\mathrm{shift}=-s,\;\dim=W) \end{array}$$

where Roll(·,shift,dim) represents a cyclic shift operation along a specified dimension, *s* is shift step, and *H* and *W* represent the height and width dimensions of the feature map, respectively. After concatenation, each spatial position aggregates information from neighboring positions across different channel subspaces, effectively expanding the receptive field.

(20) $$S_{\mathrm{BSCR}}\left(X\right)=\mathrm{Concat}\;\left([X_{1}^{\prime },\;X_{2}^{\prime },\;X_{3}^{\prime },\;X_{4}^{\prime }],\;\dim=1\right)$$

Through depth-wise separable convolution and the BSCR bidirectional channel shift mixing strategy, EUCBSC enhances spatial detail fidelity and inter-channel information flow during upsampling.

## 4. Experiments

### 4.1. Datasets

To comprehensively evaluate the performance of the proposed algorithm, this study selected three representative UAV object-detection datasets for experimental validation: VisDrone-2019 [3], UAVDT [4], and DOTA1.0 [5].

The VisDrone-2019 Dataset is a UAV vision benchmark dataset constructed by the AISKYEYE team of Tianjin University, specifically designed for object detection and tracking tasks in UAV scenarios. The dataset covers 10 object categories: pedestrian, people, bicycle, car, van, truck, tricycle, awning-tricycle, bus, and motor. It includes various scenarios, including urban, rural, highways, and construction sites, as well as complex environments with various disturbances, different weather conditions, and different object scales, providing an ideal testing platform for algorithm robustness evaluation. We divided it into a training set containing 6471 images, a validation set containing 548 images, and a test set containing 1610 images for experiments.

The UAVDT Dataset is a large-scale challenging benchmark dataset specifically designed for UAV object detection and tracking tasks. Images cover various complex urban scenarios, including squares, main roads, toll stations, highways, intersections, and T-junctions, providing important references for evaluating algorithm performance in practical applications. It contains three vehicle object categories: car, truck, and bus. Data are divided into 20,368 training samples, 8147 validation samples, and 12,220 test samples in total.

The DOTA-v1.0 Dataset is one of the most influential large-scale benchmark datasets in the field of aerial image object detection. The dataset covers 15 object categories: plane, ship, storage tank, baseball diamond, tennis court, basketball court, ground track field, harbor, bridge, large vehicle, small vehicle, helicopter, roundabout, soccer field, and swimming pool. Dataset images come from Google Earth, GF-2 satellite, JL-1 satellite, and CycloMedia B.V. aerial imagery, with diverse geographical locations, sensor types, and shooting platforms, providing a comprehensive testing environment for algorithm generalization performance evaluation. The dataset is divided into 15,749 training images and 5297 validation images.

### 4.2. Implementation Details

The experimental environment of this study adopts mainstream deep learning frameworks and hardware configurations to ensure reproducibility and comparability of experimental results. As shown in Table 1, the operating system is Ubuntu LTS 22.04. Experiments are based on PyTorch 2.0.1 and CUDA 11.7, using the Python 3.10 programming language, and conducted on a workstation equipped with NVIDIA GeForce RTX 4090 GPU (24 GB memory). The CPU uses an Intel Core(TM) i7-13700KF processor equipped with 64 GB DDR4 system memory.

The training was performed using an AdamW optimizer with an initial learning rate of $1\times 10^{-4}$, momentum of 0.9, and weight decay of $1\times 10^{-4}$. The training process adopts a cosine annealing learning-rate scheduling strategy, with total training epochs set to 300. We employed a warmup strategy with 2000 iterations. Batch size is set to 8, and input image size is uniformly resized to 640×640 pixels to ensure fair comparison among different configurations. For data augmentation, we apply random flipping with a probability of 0.5, random scaling between 0.5 and 1.5, and Mosaic augmentation with a probability of 0.5, which is disabled during the last 30 training epochs.

### 4.3. Evaluation Metrics

To comprehensively evaluate the performance of the proposed algorithm, this study adopts the COCO evaluation metric system widely used in object detection. Specific evaluation metrics include: AP50:95, representing average precision calculated over an IoU threshold range of 0.5 to 0.95 (incremented by 0.05), as the most important comprehensive performance metric; AP50: representing average precision at a 0.5 IoU threshold; APs: measuring average precision of small objects below 32×32 pixels, which is a key metric directly reflecting algorithm effectiveness on small objects in UAV images; APm: evaluating average precision of medium-sized objects between 32×32 and 96×96 pixels; APl: evaluating average precision of large objects exceeding 96×96 pixels. Computational efficiency is quantified through GFLOPS and Params (expressed in millions), measuring inference complexity and model storage requirements respectively.

### 4.4. Ablation Studies

#### 4.4.1. Effect of Multi-Scale Pooling Kernel Configuration

To verify the effectiveness of the multi-scale pooling kernel configuration in the proposed MREAM module, we conducted systematic ablation experiments on different pooling kernel combination strategies, focusing on exploring the impact of single-scale, dual-scale, triple-scale, and different four-scale configurations on aerial small object detection performance. Experimental results are shown in Table 2.

The experimental results in Table 2 verify the effectiveness and rationality of our proposed progressive multi-scale design. The single-pooling kernel configuration shows obvious limitations in detection performance, with configuration A achieving only 45.8% AP50. The dual-scale and triple-scale configurations show gradual improvement trends across metrics, but have limited capability in capturing multi-scale feature information. The five-scale configuration shows AP50 dropping to 46.2%, indicating that excessive scale branches may introduce redundant information and computational burden. Our four-scale progressive configuration achieves continuous coverage from local details to global context, better adapting to characteristics of drastic object scale changes in UAV images, achieving 47.2% AP50, small object detection accuracy APs of 20.1%, and medium object accuracy APm of 38.0%. This experimental result fully verifies that the MREAM module, through the progressive multi-scale pooling kernel combination strategy, can effectively capture multi-level feature representations of objects at different scales.

#### 4.4.2. Effect of Edge Enhancement Mechanism

To verify the effectiveness of the edge enhancement strategy in the proposed EdgeEnhancer sub-module, we conducted comparative experiments on different edge feature extraction methods. Experimental results are shown in Table 3.

The experimental results in Table 3 show that adopting edge enhancement strategies can effectively improve object detection performance. Traditional edge detection operators demonstrate certain feature enhancement capability, with the Sobel operator improving APs by 0.4% compared to the no-edge-enhancement configuration, and the Laplacian operator improving APs by 0.7%. In contrast, our proposed average pooling-based high-frequency and low-frequency decomposition edge enhancement method achieves optimal performance across all evaluation metrics, improving AP50 by 1.3% compared to baseline, APs by 1.2%, APm by 1.1%, and APl by 1.2%.

#### 4.4.3. Validation of AMFFN Feature Fusion Network Effectiveness

To verify the effectiveness of each sub-module in the proposed AMFFN feature fusion network, we designed module combination ablation experiments. Experimental results are shown in Table 4.

The experimental results in Table 4 show that both SDEC and HSAT modules make significant contributions to detection performance. Using the SDEC module alone improves AP50 by 1.3% compared to baseline, and using the HSAT module alone brings 0.9% AP50 improvement. When both modules work together, AMFFN achieves 46.8% in AP50, improving by 2.5% compared to baseline, fully verifying the overall effectiveness of the proposed AMFFN feature fusion network.

#### 4.4.4. Overall Ablation Experimental Results Analysis

**Figure 6:**
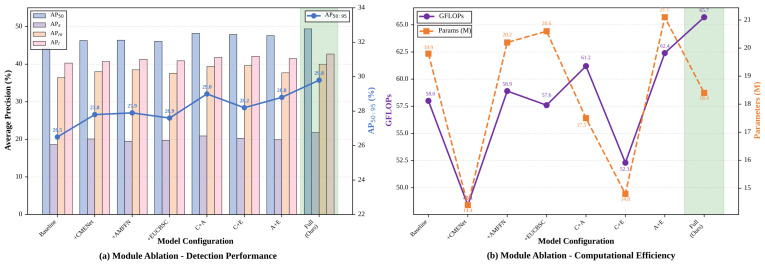
Module ablation experimental results on the VisDrone-2019 validation set. In the configuration labels, C, A, and E represent CMENet, AMFFN, and EUCBSC, respectively. (**a**) Detection performance comparison across different module configurations. (**b**) Computational efficiency comparison.

To comprehensively verify the effectiveness of each core module in EAGLE-DET and their synergistic effects, we conducted systematic overall ablation experiments. As shown in Table 5 and Figure 6, we systematically evaluated eight configuration schemes. To verify the reliability of our results, we conducted five independent training runs of the full EAGLE-DET model with different random seeds and we report the 95% confidence interval for the detection accuracy metrics.

The experimental results show that all three core modules can independently improve detection performance. The CMENet alone achieves the largest single-module improvement, with AP50 increasing by 2.9% and APs by 1.5%. Using the AMFFN feature fusion network alone brings 2.5% and 1.4% improvements in AP50 and AP50:95. Integrating the EUCBSC module alone also achieves 1.8% AP50 and 0.4% AP50:95 performance improvements.

Any dual module consistently outperforms either individual component, indicating complementarity rather than redundancy. Notably, CMENet + AMFFN performs best, improving the AP50 by 3.9% and the AP50:95 by 2.5% compared to baseline, significantly exceeding the simple addition effects of single modules. CMENet + EUCBSC improves the AP50 by 3.6% while maintaining a lightweight design. By contrast, AMFFN + EUCBSC yields 47.6% for AP50, a comparatively modest gain, as without CMENet’s edge-enriched representations, the upstream feature quality limits the effectiveness of both downstream modules.

More importantly, our complete EAGLE-DET architecture achieves optimal performance across all evaluation metrics, with AP50, AP50:95, and APs reaching 49.5%, 29.8%, and 21.8% respectively, improving 5.2%, 3.3%, and 3.2% respectively compared to baseline. This experimental result fully verifies that there exist significant synergistic enhancement effects among the three modules CMENet, AMFFN, and EUCBSC. Through cascaded collaboration of edge feature enhancement, sparse attention fusion, and spatial-channel coordination, they can achieve comprehensive optimization of cross-scale object detection performance while maintaining model lightweight function, obviously improving detection capability for small objects in aerial scenarios.

### 4.5. Comparison Experiments

#### 4.5.1. Comparison of Backbone Network Architectures

**Figure 7:**
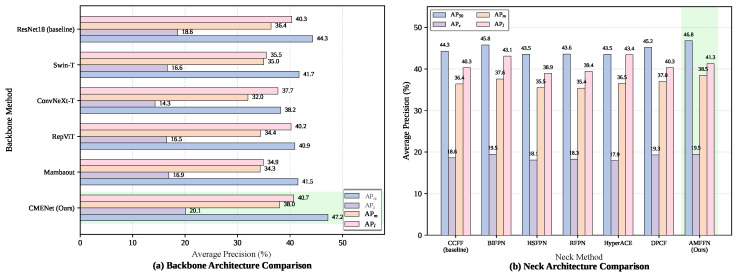
Comparison of backbone and neck network architectures. (**a**) Backbone architecture comparison. (**b**) Neck architecture comparison.

To verify the effectiveness of our proposed CMENet backbone network, we conducted backbone network architecture comparison experiments on the VisDrone-2019 validation set. The experimental results are shown in Table 6 and Figure 7.

Our proposed CMENet achieves optimal performance across all key evaluation metrics, with AP50 and AP50:95 reaching 47.2% and 27.8%, improving 2.9% and 1.3% respectively compared to baseline ResNet18, while CMENet’s parameter count is only 14.4 M, 60.7% lower than Swin-T.

#### 4.5.2. Comparison of Neck Network Architectures

To verify the effectiveness of our proposed AMFFN feature fusion network, we conducted neck network architecture comparison experiments on the VisDrone-2019 validation set. Experimental results are shown in Table 7.

Notably, our proposed AMFFN achieves the highest AP50 of 46.8% and an AP50:95 of 27.9% among all compared neck architectures. It also achieves the highest APs of 19.5% and APm of 38.5%. BIFPN and HyperACE perform better on large objects than AMFFN due to their bidirectional feature pyramid or hypergraph enhancement strategies. Nevertheless, for the core challenge of small and medium object detection in UAV imagery, AMFFN provides the most balanced and effective feature fusion.

#### 4.5.3. Upsampling Comparison Experiments

To verify the effectiveness of our proposed EUCBSC upsampling module, we conducted upsampling method comparison experiments on the VisDrone-2019 validation set. The experimental results are shown in Table 8.

Among all compared upsampling methods, our EUCBSC achieves the highest AP50 of 46.1%, AP50:95 of 26.9%, APs of 19.7%, and APm of 37.6%. However, CARAFE achieves a higher APl of 42.2% compared to our 40.9%, indicating that content-aware feature reorganization may preserve more structural information for large objects.

#### 4.5.4. Comparison with State-of-the-Art Methods

**Figure 8:**
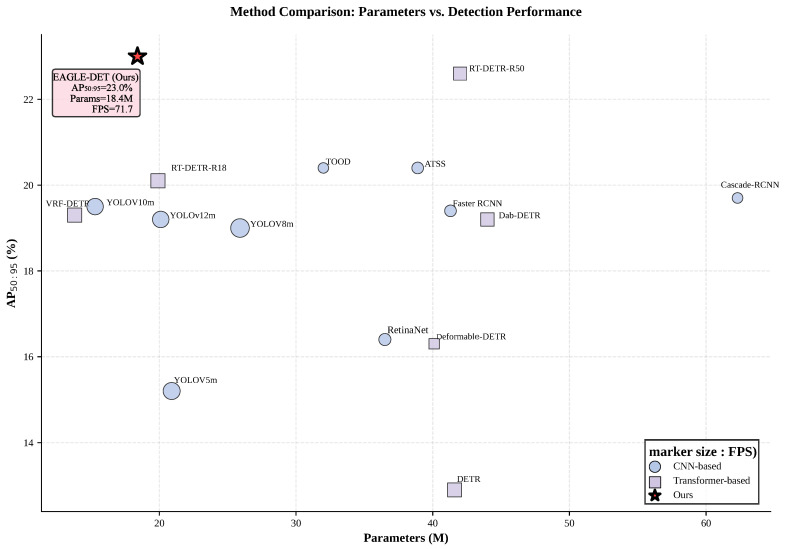
Comparison with state-of-the-art methods on VisDrone-2019 test set. The scatter plot shows the trade-off between detection performance and model parameters. Circle size represents FPS (frames per second).

To comprehensively evaluate the performance of our proposed EAGLE-DET algorithm, we selected various representative detection methods for comparison experiments, including two-stage detectors, one-stage CNN-based detectors, and Transformer-based methods. For the baseline, our method, and the strongest competing methods, we report the average accuracy accompanied by a 95% confidence interval. The experimental results are shown in Table 9 and Figure 8.

Compared to the baseline, EAGLE-DET achieves improvements of 4.5% in AP50 and 2.9% in AP50:95, with inference speed reaching 71.7 FPS. Two-stage detectors generally have large computational overhead and slow inference speed. Faster RCNN and Cascade-RCNN achieve 32.9% and 32.6% AP50 respectively, but their GFLOPS reach 208.9 and 236.6. Among CNN-based detectors, YOLOv10m and YOLOv11m achieve higher inference speeds of 105.8 and 117.1 FPS respectively, but their detection accuracy is notably lower, with AP50:95 of 19.5% for YOLOv10m and 19.5% for YOLOv11m, compared to our 23.0%. For Transformer-based detectors, DAB-DETR achieves a competitive APl of 48.3%, surpassing our 42.6%, primarily because its dynamic anchor box mechanism favors larger objects, whereas EAGLE-DET is specifically designed for small object scenarios. It should be noted that RT-DETR-R50 achieves a comparable AP50 of 39.1% versus our 39.7% and a slightly lower AP50:95 of 22.5% versus our 23.0%, but this comes at the cost of 2.3 times more parameters, specifically 42.0 million compared to our 18.4 million, and approximately 2.0 times more GFLOPs, specifically 129.6 versus our 65.7. For UAV-oriented methods, UAV-DETR-R18 and VRF-DETR both yield lower AP50, AP50:95, and APs than EAGLE-DET. These comparisons indicate that EAGLE-DET outperforms all the above methods in AP50, AP50:95, APs and APm, achieving the best balance between accuracy and efficiency.

**Figure 9:**
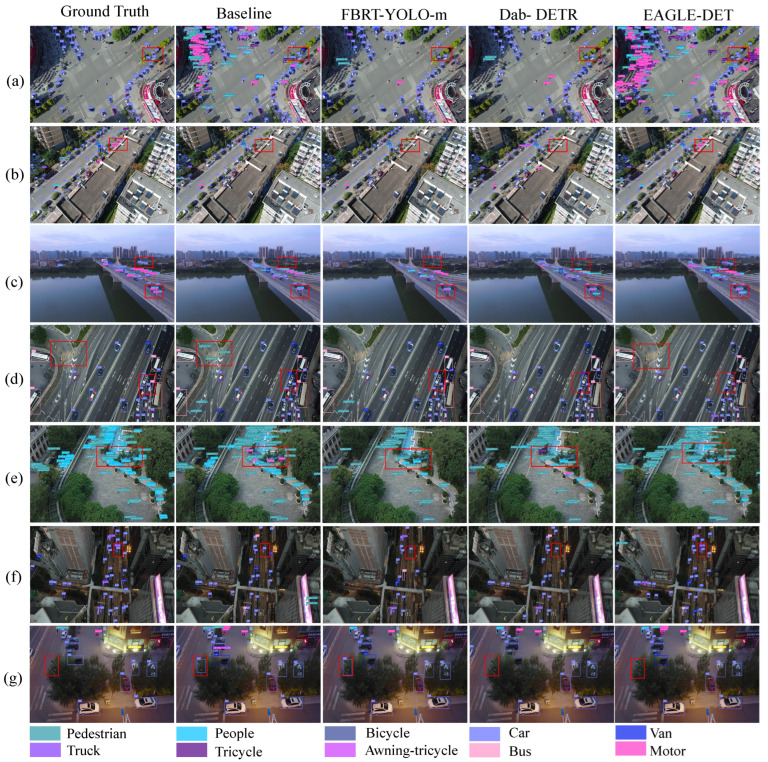
Visualization of detection results on VisDrone-2019 test set. Comparison between Ground Truth, Baseline (RT-DETR-R18), FBRT-YOLO-m, DAB-DETR, and EAGLE-DET across various challenging scenarios: (**a**) Enhanced small object identification capability in urban intersection scenes. (**b**) Accurate detection and category discrimination for ultra-small objects. (**c**) Multi-scale object detection in complex scenes with both near large vehicles and distant small motorcycles. (**d**) Fine-texture perception for ultra-small pixel objects, correcting baseline misdetections. (**e**) Dense object scene processing with precise boundary distinction. (**f**) Robust detection under extreme lighting conditions (night low-light). (**g**) Accurate identification of occluded objects while suppressing false detections. Red boxes indicate missed detections or misclassifications by baseline or other methods that are correctly handled by EAGLE-DET.

To more intuitively demonstrate EAGLE-DET’s detection performance, Figure 9 shows a visualization comparison of the VisDrone-2019 test set.

**Figure 10:**
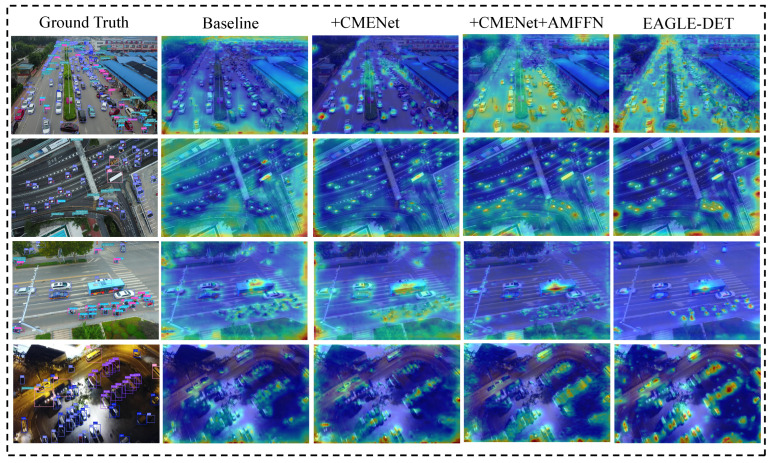
Feature activation heatmaps under different scenarios comparing Ground Truth, Baseline, CMENet + AMFFN, and EAGLE-DET. The visualization covers various challenging aerial scenarios, including dense parking lots, oblique viewing angles, multi-scale intersections, and night low-light environments.

Figure 10 shows feature activation heatmaps under different scenarios, demonstrating EAGLE-DET’s feature-learning capability. It can be observed that baseline heat response generally shows scattered and blurred characteristics, with activation regions deviating from object positions and extremely weak responses under low-light conditions. In contrast, EAGLE-DET’s heat response focuses more precisely on object regions, with clear activation boundaries highly matching actual object positions. A closer examination of the progressive heatmaps reveals interpretable feature response changes corresponding to each module’s function. After incorporating CMENet, the activations transition from spatially diffuse patterns to contour-concentrated responses, consistent with the module’s high-frequency edge enhancement mechanism that explicitly preserves high-pass filtered features. The subsequent addition of AMFFN further refines the activations by producing semantically coherent responses across different object scales, reflecting the cross-scale sparse attention’s ability to establish semantic correspondence between shallow spatial details and deep semantic representations. Finally, the complete EAGLE-DET with EUCBSC achieves the sharpest activation boundaries with minimal background leakage, demonstrating the bidirectional channel-shift mixing’s effectiveness in preserving spatial structural integrity during feature reconstruction.

### 4.6. Generalization Experiments

This paper conducted generalization experiments on the representative datasets UAVDT and DOTA1.0. The experimental results in Table 10 show that the EAGLE-DET algorithm achieves significant performance improvements on both representative datasets. On the UAVDT dataset, compared to baseline algorithm RT-DETR-R18, EAGLE-DET improves AP50:95 by 2.2%, APs by 3.2%, and APm by 1.9% respectively. On the more challenging DOTA1.0 remote sensing dataset, EAGLE-DET also demonstrates superior performance, with AP50:95 reaching 50.6%, improving 1.5% compared to baseline.

We also present fine-grained category performance comparison on the DOTA dataset in Table 11. EAGLE-DET achieves significant improvements in multiple categories, with especially notable improvements in small-scale object categories: small_vehicle’s AP50 improves from 67.8% to 69.2%, large_vehicle’s AP50 improves from 85.3% to 87.3%, and helicopter’s AP50 improves from 53.8% to 69.9%.

**Figure 11:**
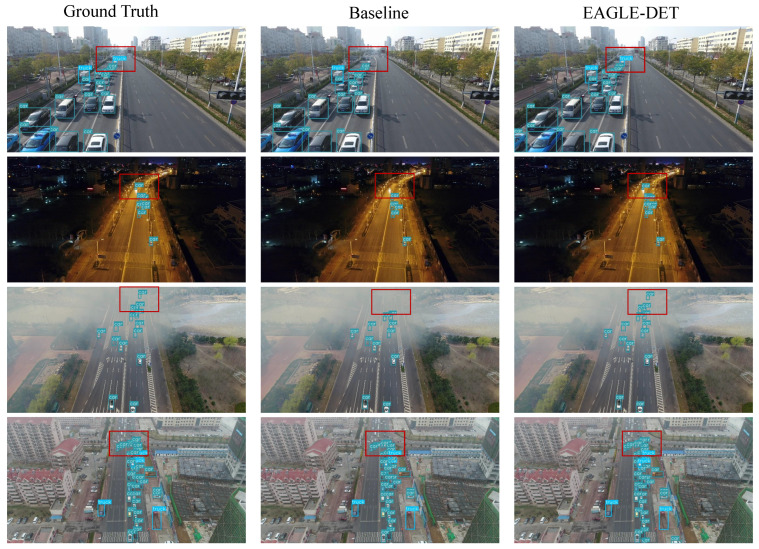
Visualization of detection results on the UAVDT dataset. Comparison between Ground Truth, Baseline (RT-DETR), and EAGLE-DET across various traffic scenarios including highways, intersections, and urban roads. EAGLE-DET demonstrates superior detection capability for small and distant vehicles under different viewing angles and lighting conditions. Red boxes highlight improved detections compared to baseline.

**Figure 12:**
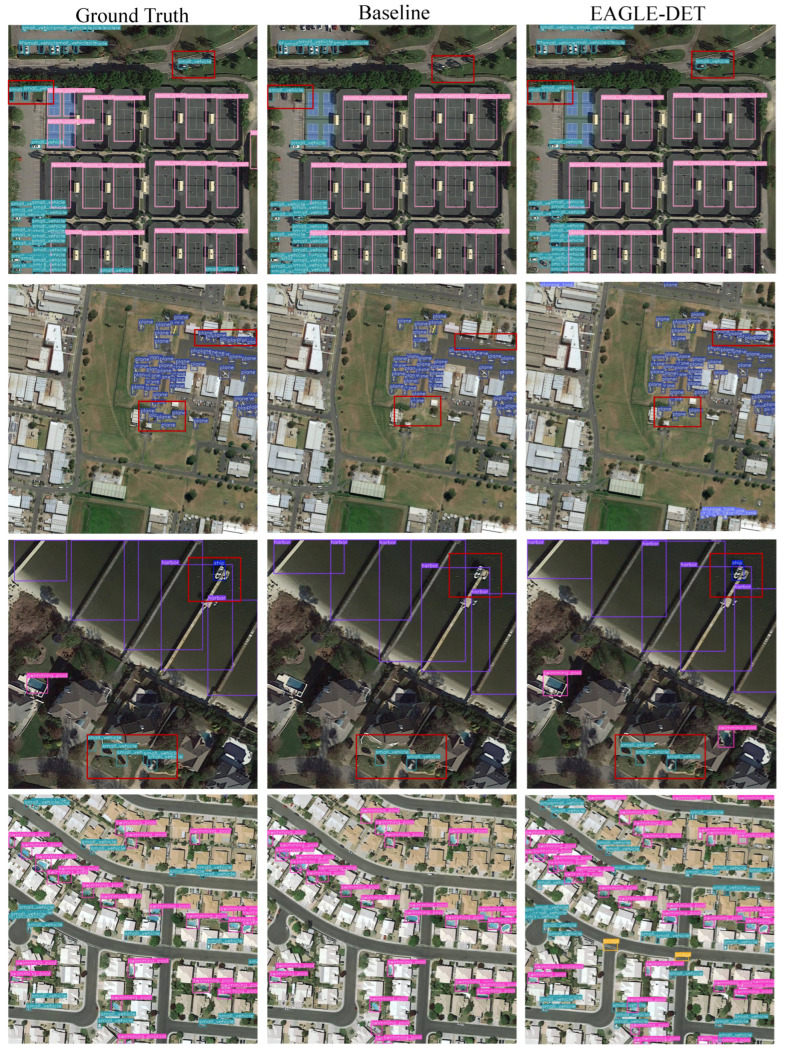
Visualization of detection results on the DOTA1.0 dataset. Comparison shows EAGLE-DET’s performance on various object categories in remote sensing imagery including storage tanks, ships, harbors, and aircraft. Red boxes highlight improved detections compared to baseline.

Figure 11 and Figure 12 show detection result visualizations on UAVDT and DOTA1.0 datasets respectively.

## 5. Discussion

EAGLE-DET addresses UAV small object detection through three modules: CMENet targets edge attenuation via high-frequency decomposition, AMFFN resolves semantic conflict via cross-scale sparse attention, and EUCBSC recovers detail loss via bidirectional channel-spatial reorganization. This design ensures each module produces higher-quality inputs for subsequent stages, forming a coherent repair chain.

It is worth noting that EAGLE-DET achieves these improvements while maintaining a favorable complexity–performance trade-off. Compared to the RT-DETR-R18 baseline with 19.9 M parameters and 58.0 GFLOPs, EAGLE-DET reduces parameters to 18.4 M (7.5% reduction) and maintains real-time inference at 71.7 FPS, demonstrating that the three-module design introduces targeted complexity rather than indiscriminate architectural expansion. The CMENet backbone alone achieves 16.6% GFLOPs reduction and 27.6% parameter reduction while improving AP50 by 2.9%, indicating that our edge-aware design replaces rather than supplements the original feature extraction overhead. Furthermore, in the context of UAV small object detection where targets occupy as few as tens of pixels, improvements of 3.2% in APs and 5.2% in AP50 on the VisDrone-2019 validation set represent meaningful advances, as each percentage point requires recovering discriminative features from extremely sparse pixel representations.

While our approach demonstrates significant improvements, several important limitations warrant discussion. First, the AMFFN and EUCBSC modules, while effective for cross-scale semantic alignment and high-fidelity feature reconstruction, introduce non-negligible computational overheads. The pyramidal sparse attention computation within the HSAT module of AMFFN involves coarse-grained and fine-grained two-level attention operations across multi-scale features, and the EUCBSC module’s cascaded depth-wise separable convolution, channel shuffle, and BSCR operations further increase computational costs during the upsampling stage. As shown in the ablation experiments (Table 5), the combination of AMFFN and EUCBSC raises GFLOPs from 58.0 to 62.4, and the complete EAGLE-DET configuration reaches 65.7 GFLOPs. Current GPU implementations are not fully optimized for our specific sparse attention and bidirectional channel shift operation patterns, suggesting the potential for more efficient attention mechanisms or lightweight upsampling alternatives in future work.

Second, the fine-grained category analysis on the DOTA1.0 dataset (Table 11) reveals uneven performance gains across different object categories. While EAGLE-DET achieves notable improvements for categories such as helicopter (+16.1% AP50) and swimming pool (+2.9% AP50), it shows degradation for soccer field (−4.1% AP50), roundabout (−2.7% AP50), and basketball court (−14.2% AP50). These categories typically feature large, relatively uniform regions where our edge enhancement strategy may introduce unnecessary high-frequency noise, suggesting that the edge-centric design philosophy, while beneficial for small, compact objects, may not universally benefit all object types. This observation indicates the need for category-adaptive feature processing strategies.

Third, our architecture exhibits diminishing returns at very high resolutions. While the current 640×640 input resolution achieves a good accuracy–efficiency balance, scaling to higher resolutions for high-altitude drone footage would cause memory requirements to grow quadratically in the HSAT module due to the attention computation, creating deployment challenges on memory-constrained edge devices. The SDEC module’s space-depth transformation with the stride-2 and stride-4 branches further amplifies channel dimensions at higher resolutions, potentially exceeding the memory capacity of typical UAV-mounted computing platforms.

Future work will focus on addressing these limitations through more efficient sparse attention and upsampling designs, category-adaptive feature modulation, scalable high-resolution processing, and further optimization for edge deployment, particularly targeting dedicated neural processing hardware for UAV platforms.

## 6. Conclusions

This paper proposes an improved detection method EAGLE-DET, targeting the core bottleneck problem of feature degradation in UAV aerial image small object detection. Through in-depth analysis of the degradation mechanism of small object features during deep network forward propagation, this paper systematically identifies three key degradation stages of edge attenuation, semantic conflict, and detail loss, and designs targeted cascaded feature repair strategies. The main technical contributions of this paper are summarized as follows: (1) Proposes the CMENet backbone network, which reduces computational complexity while improving small object detection accuracy through cross-stage pyramidal structure and multi-resolution edge enhancement modules; (2) Designs the AMFFN feature fusion network, which achieves adaptive semantic alignment of cross-scale features through a pyramidal sparse attention mechanism and multi-scale spatial decoupling strategy, effectively suppressing the semantic conflict problem; (3) Constructs the EUCBSC module, which enhances feature reconstruction quality through a bidirectional channel-shift mixing mechanism, effectively alleviating the upsampling detail loss problem. Comprehensive experimental validation on three representative datasets VisDrone-2019, UAVDT, and DOTA1.0 demonstrates the effectiveness of the proposed method. EAGLE-DET provides new theoretical perspectives and technical solutions for aerial small object detection algorithm design.

## Figures and Tables

**Table 1:** Experimental system environment.

Item	Model/Parameters
Operating System	Ubuntu LTS 22.04
Programming Language	Python 3.10
CPU	Intel Core(TM) i7-13700KF (Intel Corporation, Santa Clara, CA, USA)
Graphics card	NVIDIA GeForce RTX 4090 GPU (NVIDIA Corporation, Santa Clara, CA, USA)
GPU Memory	24G

**Table 2:** Multi-scale pooling kernel configuration comparison experiments.

Config	Type	Pooling Kernel Config	AP50	APs	APm
A	Single-scale	{3×3}	45.8	18.1	35.2
B	Dual-scale	{3×3,6×6}	46.0	19.2	36.4
C	Triple-scale	{3×3,6×6,9×9}	46.0	19.8	36.1
D	Four-scale	{3×3,3×3,3×3,3×3}	46.2	18.5	35.8
E	Four-scale	{6×6,6×6,6×6,6×6}	46.3	19.1	36.2
F	Four-scale	{9×9,9×9,9×9,9×9}	46.3	19.3	36.6
G	Four-scale	{12×12,12×12,12×12,12×12}	**47.6**	18.9	36.0
H	Four-scale Progressive (Ours)	{3×3,6×6,9×9,12×12}	47.2	**20.1**	**38.0**
I	Five-scale	{3×3,6×6,9×9,12×12,15×15}	46.2	19.2	36.8

Bold values indicate the best performance.

**Table 3:** Edge enhancement method comparison experiments (%).

Edge Enhancement Method	AP50	APs	APm	APl
MREAM (w/o EdgeEnhancer)	45.9	18.9	36.9	39.5
Sobel operator	46.0	19.3	37.6	40.2
Laplacian operator	45.8	19.6	37.6	40.1
AvgPool-based (Ours)	**47.2**	**20.1**	**38.0**	**40.7**

Bold values indicate the best performance.

**Table 4:** AMFFN module component ablation experiments (%).

Config	SDEC	HSAT	AP50	APs	APm
Baseline			44.3	18.6	36.4
Only SDEC	✔		45.6	19.3	37.6
Only HSAT		✔	45.2	19.0	36.9
AMFFN (Ours)	✔	✔	**46.8**	**19.5**	**38.5**

Bold values indicate the best performance.

**Table 5:** EAGLE-DET algorithm module performance analysis on VisDrone-2019 validation set (%).

Model	C	A	E	GFLOPS	Params (M)	AP50	AP50:95	APs	APm	APl
1. baseline				58.0	19.9	44.3	26.5	18.6	36.4	40.3
2	✔			48.4	14.4	47.2	27.8	20.1	38.0	40.7
3		✔		58.9	20.2	46.8	27.9	19.5	38.5	41.3
4			✔	57.6	20.6	46.1	26.9	19.7	37.6	40.9
5	✔	✔		61.2	17.5	48.2	29.0	20.9	39.4	41.8
6	✔		✔	52.3	14.8	47.9	28.2	20.3	39.6	42.1
7		✔	✔	62.4	21.1	47.6	28.8	19.9	37.7	41.5
8. Ours	✔	✔	✔	65.7	18.4	**49.5 ± 0.2**	**29.8 ± 0.1**	**21.8 ± 0.2**	**40.1 ± 0.2**	**42.5 ± 0.3**

C—CMENet; A—AMFFN; E—EUCBSC. Bold values indicate the best performance.

**Table 6:** Backbone network architecture comparison experiments on VisDrone-2019 validation set.

Backbone	GFLOPs	Params (M)	AP50	AP50:95	APs	APm	APl
Resnet18 (baseline)	58.0	19.9	44.3	26.5	18.6	36.4	40.3
Swin-T [6]	98.4	36.6	41.7	24.6	16.6	35.0	35.5
ConvNeXt-T [7]	**33.3**	**12.6**	38.2	22.2	14.3	32.0	37.7
RepVit [8]	38.3	13.6	40.9	24.3	16.5	34.4	40.2
Mambaout [37]	41.9	15.9	41.5	24.5	16.9	34.3	34.9
CMENet (Ours)	48.4	14.4	**47.2**	**27.8**	**20.1**	**38.0**	**40.7**

Bold values indicate the best performance.

**Table 7:** Neck architecture comparison experiments on VisDrone-2019 validation set.

Method	GFLOPs	Params (M)	AP50	AP50:95	APs	APm	APl
CCFF (baseline)	58.0	19.9	44.3	26.5	18.6	36.4	40.3
BIFPN [9]	64.3	20.3	45.8	27.4	**19.5**	37.6	43.1
HSFPN [38]	58.1	20.7	43.5	25.8	18.1	35.5	38.9
RFPN [39]	**56.4**	**19.6**	43.6	26.0	18.3	35.4	39.4
HyperACE [40]	60.4	21.9	43.5	26.1	17.9	36.5	**43.4**
DPCF [41]	57.0	19.9	45.2	27.1	19.3	37.0	40.3
**AMFFN (Ours)**	58.9	20.2	**46.8**	**27.9**	**19.5**	**38.5**	41.3

Bold values indicate the best performance.

**Table 8:** Upsampling comparison experiments on VisDrone-2019 validation set.

Method	AP50	AP50:95	APs	APm	APl
CARAFE [11]	44.7	26.7	18.7	36.9	**42.2**
DySample [12]	44.5	26.5	18.3	36.7	42.1
EUCB [42]	44.6	26.5	19.0	35.8	38.1
Converse2D [43]	44.0	26.4	18.5	36.0	40.8
EUCBSC (Ours)	**46.1**	**26.9**	**19.7**	**37.6**	40.9

Bold values indicate the best performance.

**Table 9:** Performance of different methods on VisDrone-2019 Test dataset (%).

Method	AP_50_	AP_50:95_	AP_*s*_	AP*_m_*	AP*_l_*	GFLOPS	Params (M)	FPS
Two-stage Object Detector								
Faster RCNN [44]	32.9	19.4	9.5	30.9	42.3	208.9	41.3	53.1
Cascade-RCNN [45]	32.6	19.7	9.9	30.9	40.6	236.6	62.3	44.3
One-stage Object Detector								
CNN-based								
GFL [46]	32.1	19.3	9.4	30.0	40.9	205.8	32.3	50.5
ATSS [47]	33.8	20.4	10.0	31.7	46.5	110.4	38.9	53.6
TOOD [48]	33.9	20.4	10.2	31.7	40.3	199.1	32.0	43.9
RetinaNet [49]	27.6	16.4	6.0	27.4	42.5	210.6	36.5	57.2
YOLOv5m [50]	28.8	15.2	7.3	23.3	30.6	48.0	20.9	113.5
YOLOv8m [51]	33.2	19.0	9.0	29.4	41.7	78.7	25.9	**135.7**
YOLOv10m [52]	34.5	19.5	9.7	30.0	41.4	58.9	15.3	105.8
YOLOv11m [53]	33.9	19.5	9.2	30.1	42.6	67.7	20.0	117.1
YOLOv12m [54]	33.6	19.2	9.4	29.8	38.6	67.2	20.1	106.4
FBRT-YOLO-m [55]	34.4	19.6	9.4	30.9	42.1	58.7	**7.4**	104.8
Transformer-based								
DETR [56]	25.8	12.9	5.6	23.4	30.2	96.5	41.6	26.8
Deformable-DETR [57]	30.0	16.3	8.7	25.4	33.0	193.0	40.1	32.1
Conditional-DETR [58]	30.4	16.2	7.9	24.4	45.8	101.3	43.5	48.5
DAB-DETR [59]	37.2	19.2	10.7	29.3	**48.3**	102.9	44.0	50.9
VRF-DETR [13]	33.9	19.3	10.7	28.1	41.1	**45.7**	13.8	79.6
RT-DETR-R34 [18]	37.6	21.8	12.8	29.6	29.4	90.0	20.1	62.4
RT-DETR-R50 [18]	39.1 ± 0.2	22.5 ± 0.1	13.2 ± 0.1	32.6 ± 0.2	42.2 ± 0.2	129.6	42.0	67.7
UAV-DETR-R18 [14]	37.4 ± 0.3	20.3 ± 0.2	12.3 ± 0.2	30.4 ± 0.2	41.5 ± 0.3	73.9	21.6	64.3
RT-DETR-R18 (Baseline) [18]	35.2 ± 0.2	20.1 ± 0.2	11.4 ± 0.2	28.9 ± 0.3	36.1 ± 0.2	57.0	19.9	78.4
EAGLE-DET (Ours)	**39.7 ± 0.2**	**23.0 ± 0.2**	**13.5 ± 0.2**	**33.5 ± 0.1**	42.6 ± 0.3	65.7	18.4	71.7

Bold values indicate the best performance.

**Table 10:** Experimental results on UAVDT and DOTA datasets.

Datasets	Method	Parameters	GFLOPS	AP50:95	APs	APm
UAVDT	RT-DETR-R18	19.9	**58.9**	84.9	76.0	87.9
	EAGLE-DET	**18.3**	65.4	**87.1**	**79.2**	**89.8**
DOTA1.0	RT-DETR-R18	19.9	**58.0**	49.1	25.8	53.0
	EAGLE-DET	**18.3**	65.5	**50.6**	**27.7**	**55.1**

Bold values indicate the best performance.

**Table 11:** Results of different categories on the DOTA datasets.

Class	RT-DETR-R18		EAGLE-DET	
	AP50	AP50:95	AP50	AP50:95
small_vehicle	67.8	42.1	**69.2**	**43.1**
large_vehicle	85.3	**76.1**	**87.3**	73.8
plane	91.9	69.4	**92.5**	**69.9**
storage_tank	74.1	45.4	**74.3**	**45.8**
ship	**87.1**	**62.2**	86.7	62.1
harbor	82.6	47.8	**83.4**	**49.0**
ground_track_field	64.0	41.5	**64.7**	**44.3**
soccer_ball_field	**60.9**	**43.9**	56.8	41.1
tennis_court	**92.5**	**84.3**	91.9	83.6
swimming_pool	60.0	25.9	**62.9**	**28.3**
baseball_diamond	**74.9**	**44.8**	72.2	44.0
roundabout	**55.7**	**31.6**	53.0	28.1
basketball_court	**59.5**	**47.4**	45.3	35.6
bridge	49.1	23.3	**52.6**	**25.9**
helicopter	53.8	32.4	**69.9**	**46.1**

Bold values indicate the best performance.

## Data Availability

The raw data supporting the conclusions of this article will be made available by the authors on request.

## Abbreviations

The following abbreviations are used in this manuscript:

UAV	Unmanned Aerial Vehicle
CMENet	Cross-stage Multi-resolution Edge Enhancement Network
AMFFN	Attention-guided Multi-scale Feature Fusion Network
EUCBSC	Enhanced Upsampling with Channel Bridging and Spatial Coordination
CSPMEE	Cross-Stage Pyramidal Multi-Resolution Edge Enhancement
MREAM	Multi-Resolution Edge Amplification Module
HSAT	Hierarchical Sparse Attention Transformer
SDEC	Spatial Decomposition Enhanced Convolution
SAFB	Sparse Attention Fusion Block
BSCR	Bidirectional Spatial Channel Reorganization
FPN	Feature Pyramid Network
NMS	Non-Maximum Suppression
AP	Average Precision
FPS	Frames Per Second

## Appendix A. Notation Summary

**Table A1:** Summary of mathematical symbols and notations.

Symbol	Module	Description
*X*	General	Input feature tensor
$\psi_{1\times 1}$, $\phi_{1\times 1}$	General	1×1 convolution mapping functions
$\phi_{3\times 3}$	General	3×3 convolution mapping function
C[·]	General	Feature concatenation operation
I(·,H×W)	General	Bilinear interpolation upsampling operation
σ	General	Sigmoid activation function
δ	General	ReLU activation function
⊙	General	Element-wise multiplication
$X_{\mathrm{bypass}}$, $X_{\mathrm{branch}}$	CMENet	Bypass and enhancement branch feature tensors
$M_{\mathrm{MREAM}}^{\left(i\right)}$	CMENet	The *i*-th MREAM unit
$P_{k\times k}^{\mathrm{adaptive}}$	CMENet	Adaptive average pooling of scale k×k
$D_{3\times 3}$	CMENet	3×3 depth-wise separable convolution
E(·)	CMENet	EdgeEnhance enhancement function
$H_{\mathrm{high}}$	CMENet	High-pass filtering result
$P_{3\times 3}^{\mathrm{avg}}$	CMENet	3×3 average pooling for low-frequency extraction
φ	CMENet	Edge feature transformation convolution
$X_{\mathrm{shallow}}$, $X_{\mathrm{deep}}$	AMFFN	Shallow and deep feature maps
$S_{\mathrm{SDEC}}$	AMFFN	Spatial decoupling convolution transformation
$T_{\mathrm{HSAT}}$	AMFFN	HSAT processing function
$A_{\mathrm{adaptive}}$	AMFFN	Adaptive feature aggregation operation
$B_{\mathrm{SAFB}}^{\left(i\right)}$	AMFFN	The *i*-th Sparse Attention Fusion Block
$A_{\mathrm{PSAttn}}$	AMFFN	Pyramidal sparse attention function
*Q*, *K*, *V*	AMFFN	Query, key, and value matrices
$S_{\mathrm{SPD}}^{\left(s\right)}$	AMFFN	Space-depth convolution with stride *s*
SE(·)	AMFFN	Squeeze-excitation attention
GAP	AMFFN	Global average pooling
$W_{1}$, $W_{2}$	AMFFN	SE weight matrices for reduction and expansion
$U_{\mathrm{bilinear}}$	EUCBSC	Bilinear upsampling operation
$D_{\mathrm{DW}}$	EUCBSC	Depth-wise separable convolution transformation
$S_{\mathrm{shuffle}}$	EUCBSC	Channel shuffle function
$S_{\mathrm{BSCR}}$	EUCBSC	Bidirectional Spatial Channel Reorganization
Roll(·)	EUCBSC	Cyclic shift operation along specified dimension
*s*	EUCBSC	Shift step in BSCR
